# Addressing Gaps in the Hypertension and Diabetes Care Continuum in Rural Bangladesh Through Digital Technology Supported Decentralized Primary Care: Study Protocol and Baseline Results for a Hybrid Effectiveness-Implementation Trial

**DOI:** 10.2196/71696

**Published:** 2026-02-02

**Authors:** Wubin Xie, Sabrina Ahmed, Ali Ahsan, Ananya Gupta, Anais Masako Keenan, Tanmoy Sarker, Fahmida Akter, Aysha Anan, Md Mokbul Hossain, Zahidul Quayyum, AHM Enayet Hussain, Robed Amin, Imran Ahmed Chowdhury, Mithila Faruque, Sohel Reza Choudhury, Ian Y Goon, Fred Hersch, Lora L Sabin, Brian Oldenburg, John Chambers, Malay Kanti Mridha

**Affiliations:** 1 Population and Global Health, Lee Kong Chian School of Medicine, Nanyang Technological University, Singapore, Singapore; 2 Department of Public Health, North South University, Dhaka, Bangladesh; 3 NCD Epidemiology Research Center, Shiga University of Medical Science, Otsu, Shiga, Japan; 4 Centre for Non-communicable Diseases and Nutrition, BRAC James P Grant School of Public Health, BRAC University, 65, Bir Uttam AK Khandakar Road, Mohakhali, Dhaka, Bangladesh, 880 248812213 ext 18; 5 Australian Institute of Family Studies, Melbourne, Australia; 6 Department of Health Promotion, Education, and Behavior, Arnold School of Public Health, University of South Carolina, Columbia, SC, United States; 7 BRAC James P Grant School of Public Health, BRAC University, Dhaka, Bangladesh; 8 Non-Communicable Disease Control, Directorate General of Health Services, Dhaka, Bangladesh; 9 BRAC Health Programme, BRAC, Dhaka, Bangladesh; 10 Department of Noncommunicable Diseases, Bangladesh University of Health Sciences, Dhaka, Bangladesh; 11 Department of Epidemiology and Research, National Heart Foundation Hospital and Research Institute, Dhaka, Bangladesh; 12 Baker Heart and Diabetes Institute, Melbourne, Australia; 13 Tyree Foundation Institute of Health Engineering, UNSW Sydney, Sydney, Australia; 14 Sprightly Pte Ltd, Singapore, Singapore; 15 Google Health, Palo Alto, CA, United States; 16 Department of Global Health, School of Public Health, Boston University, Boston, MA, United States; 17 School of Psychology and Public Health, La Trobe University, Melbourne, Australia; 18 Department of Epidemiology and Biostatistics, School of Public Health, Imperial College London, London, United Kingdom

**Keywords:** hypertension, diabetes, decentralization, task shifting, Bangladesh, Simple app

## Abstract

**Background:**

Hypertension and diabetes are very common, interrelated chronic conditions. Awareness, diagnosis, treatment, and control rates of these conditions remain low, and access to quality care—particularly in rural areas—is a persistent challenge in many low- and middle-income countries. Strengthening primary health care, including the use of digital tools, is important to improve management of these chronic conditions.

**Objective:**

This study aims to assess the implementation and effectiveness of a multicomponent, decentralized primary care model in comparison with a digital health–only intervention and usual care in rural Bangladesh.

**Methods:**

The study applies a type 2 hybrid effectiveness-implementation design, using a 3-arm quasi-experimental approach, comprising 2 intervention arms and 1 usual care comparison arm. The study is being conducted across 3 subdistricts in the Dinajpur district, Rangpur division, northern Bangladesh. Primary outcomes include blood pressure and blood glucose control rates, assessed by population-based repeated cross-sectional surveys with independent samples, supplemented by facility-based prospective cohort data. Additionally, a mixed methods process evaluation is being conducted to capture the quantity, fidelity, adaptations, reach, and context of the interventions.

**Results:**

The baseline community survey was conducted between January and March 2024, enrolling 6849 participants distributed across 3 arms: 2262 in usual care, 2287 in the digital-only arm, and 2300 in the multicomponent intervention arm. Participants had a mean age of 55.9 (SD 10.6) years with equal sex distribution (female: 3432/6849, 50.1%). Educational attainment was low, with 39.5% (2704/6849) of participants having no formal schooling and only 12.1% (917/6849) attaining secondary or higher education. The majority (6316/6849, 92.2%) reported being either self-employed or homemakers. The age-standardized baseline blood pressure control rate among all participants with hypertension was 10.2% overall, while the glycemic control rate among those with diabetes was 14.9%. Awareness and treatment rates for hypertension were 35.3% and 23.0%, respectively, compared to 60.7% and 34.5% for diabetes.

**Conclusions:**

The study findings will provide critical evidence on scalable models for decentralized noncommunicable disease care and will have important implications for improving the management of hypertension and diabetes in Bangladesh and similar low-resource settings globally.

## Introduction

Hypertension is the leading modifiable risk factor for cardiovascular diseases (CVDs) [1,2], affecting approximately 1 in 5 adult women and 1 in 4 adult men globally [3]. Between 1990 and 2019, the number of adults with elevated blood pressure (BP) has doubled from 650 million to 1.3 billion, largely driven by the increasing prevalence in low- and middle-income countries (LMICs) [4]. Diabetes affects 10.5% of adults worldwide, with nearly half of cases undiagnosed [1]. Poorly controlled hypertension and hyperglycemia contribute substantially to complications including heart disease, stroke, chronic kidney disease, and vision loss [1,5], as well as premature mortality [6]. Despite these health risks, the rates of awareness, treatment, and control of hypertension and diabetes remain alarmingly low in LMICs [7-9], where health systems, especially at the primary care level [10], struggle to meet the rising burden of noncommunicable diseases (NCDs) [11-13]. In 2022, the 75th World Health Assembly established the first global coverage targets for diabetes, aiming for 80% diagnosis rates among people with diabetes and 80% glycemic control among diagnosed cases by 2030. According to the World Health Organization (WHO) 2023 Global Report on Hypertension, achieving the 80-80-80 targets could prevent approximately 79 million nonfatal myocardial infarctions and 76 million cardiovascular deaths worldwide [14].

Access to NCD care remains severely constrained in the primary care systems of most LMICs, particularly in rural settings where health facilities often lack capability and capacity for NCD management [15,16]. Once diagnosed, patient retention poses a tremendous challenge for hypertension and diabetes care [17,18]. Studies in LMIC report dropout rates of over 50% of enrolled patients 6 months following treatment initiation [19,20]. Decentralizing care by task shifting (or task sharing) has been shown to be a feasible and acceptable strategy for scaling up antiretroviral therapy for HIV and AIDS care in resource-constrained settings [21-23]. This approach, which involves transferring routine management of stable patients from physician-managed clinics to peripheral facilities staffed by nonphysician health workers, enhances geographic accessibility, reduces patient costs, and enables the primary care system to serve a much larger patient population [21]. Despite these demonstrated benefits and the potential to address health care workforce shortages and improve access to HIV and AIDS care, few studies have evaluated this strategy for NCDs. Several small-scale studies have experimented with this approach [24-26], including the India Hypertension Control Initiative and the integrated tracking, referral, electronic decision support, and care coordination program for the management of hypertension and diabetes in India [22]. However, broader implementation and effectiveness remain understudied, and the impact on awareness and treatment remains largely unknown.

While digital tools for improving hypertension control and diabetes management are increasingly being tested in LMICs, the evidence for their effectiveness is mixed [27-29], and it is unclear whether standalone digital solutions can significantly improve NCD care delivery [30]. In contrast, multicomponent interventions in primary care have been shown to improve treatment outcomes in several large-scale randomized controlled trials [28,31-33]. These interventions typically combine digital tools with health care provider training, task shifting, community health worker (CHW)-led home-based BP monitoring, counseling, and referral. While showing effectiveness as a whole, partitioning the effect of individual intervention components was not possible in these studies. Nevertheless, evaluating the components individually, early evidence from LMICs shows that task shifting, when accompanied by health system restructuring, is a potentially effective strategy for improving access to NCD care [34], and that using CHWs in health programs may be effective in BP and diabetes control [35].

Among emerging digital solutions, the Simple app—developed and maintained by Resolve to Save Lives—has been increasingly used in South Asia and other LMICs [36]. Currently deployed in 7245 public health facilities in Bangladesh, Ethiopia, India, and Sri Lanka, the platform has registered 3.9 million people with hypertension, diabetes, or both conditions [37,38]. The app addresses two critical determinants of treatment success: (1) timely medication titration and (2) therapy adherence and continuity [39]. Its design intentionally limits data collection to essential variables (eg, prescriptions, follow-up visits, and BP or blood glucose [BG] measurements) to optimize usability in busy primary care settings. However, rigorous evaluation of its impact on clinical and behavioral outcomes remains pending [37].

As part of a UK National Institute for Health Research–funded program, we previously conducted a proof-of-concept trial demonstrating the feasibility of a digital tool to support decentralized care for hypertension and diabetes management in rural Bangladesh [26]. Building on these promising findings, we designed a 3-arm hybrid implementation-effectiveness trial to evaluate the impact of the Simple app within a multicomponent intervention framework, addressing critical gaps in understanding both effectiveness in clinical outcomes and implementation challenges. The paper describes the study methods and baseline findings, serving as a reference framework for subsequent publications from the Dinajpur study.

## Methods

### Study Setting and Populations

Bangladesh, an LMIC in South Asia with a total population of 171.5 million, has undergone major demographic and epidemiologic transitions in recent decades, and NCDs now account for approximately 70% of all deaths nationally [40]. The prevalence of hypertension and diabetes among Bangladeshi adults is estimated to be 27.4% and 9.8%, respectively [41,42]. The awareness, treatment, and control rates among people living with these conditions remain alarmingly low [41,42]. Furthermore, tobacco use, insufficient fruit or vegetable intake, and overweight are highly prevalent [43]. Over the past decade, the Government of Bangladesh has tried to implement national multisectoral actions to strengthen NCD care, most notably through the establishment of dedicated NCD care delivery points (known as “NCD Corners”) in subdistrict hospitals (“Upazila health complex” in local language) since 2011. The initiative’s planned decentralization to village-level primary care facilities (community clinics [CCs]) has not materialized [41]. Consequently, access to public NCD services remains severely limited in rural Bangladesh [41]. The Simple app has been progressively implemented across primary NCD care facilities in Bangladesh since 2020. As of current reporting, more than 180 facilities use the platform to manage over 350,000 hypertension and diabetes patients. The app’s integration with the national health management information system enables real-time performance monitoring and feedback for health facilities.

### Study Design

This hybrid effectiveness-implementation trial simultaneously evaluates intervention effectiveness and implementation strategies [44], using a 3-arm, quasi-experimental design. The aims of this trial are (1) to evaluate the effectiveness of a multicomponent, decentralized care model for hypertension and diabetes management within the public primary care systems, compared to both usual care and a standalone digital health intervention (Simple app); (2) to examine implementation processes, including explanatory factors influencing intervention effectiveness and barriers to and facilitators of delivery and sustainability; and (3) to undertake an economic evaluation. We hypothesize that compared with usual care, the multicomponent decentralized primary care—supported by the Simple app—will improve all steps along the hypertension and diabetes care continuum. Conversely, we hypothesize that the mobile health intervention alone (Simple app) may improve BP and glycemic control compared with usual care but will have a limited impact on rates of screening, diagnosis, and treatment; multicomponent integrated care will lead to a higher treatment success rate compared to the mobile health intervention alone.

**Figure 1.**
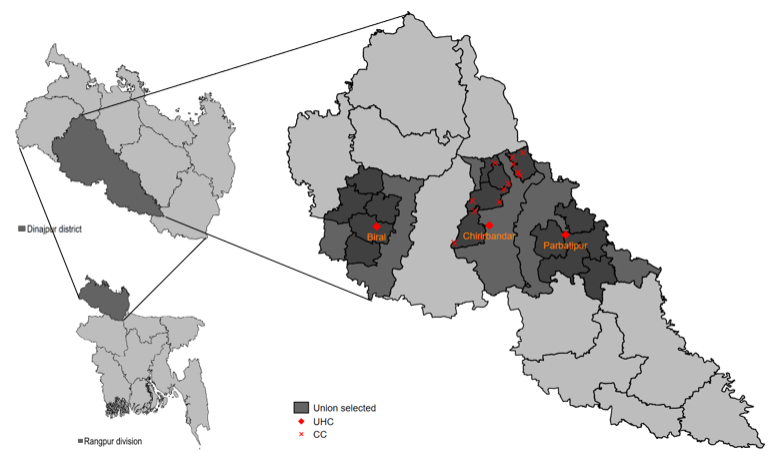
Study sites: Dinajpur district, Rangpur division, Bangladesh. Multicomponent interventions are implemented in the Chirirbandar subdistrict, a digital-only intervention is implemented in the Parbatipur subdistrict, and the Biral subdistrict is the usual care arm. CC: community clinic; UHC: Upazila health complex.

The study is being conducted across 3 subdistricts in the Dinajpur district, Rangpur division, northern Bangladesh (Figure 1 and Table 1). The multicomponent intervention is being implemented in the Chirirbandar subdistrict, while the digital health–only intervention is being implemented in the Parbatipur subdistrict. The Biral subdistrict serves as the reference site. Primary outcomes include BP and glycemic control rates, assessed by population-based repeated cross-sectional surveys with independent samples, supplemented by facility-based prospective cohort data. Additionally, a mixed methods process evaluation is being conducted to capture the quantity, fidelity, adaptations, reach, and context of the interventions. The study duration is 36 months, including an intervention period of 24 months.

**Table 1.** Population and health facility statistics in study areas.^a^

Characteristics	Study sites		
	Chirirbanda (multicomponent)	Parbatipur (digital health only)	Biral (usual care)
Area (square km)	312.7	395.0	353.4
Population, n	292,500	365,103	257,925
Adults aged 40+ years, %	25.6	25.4	25.3
Persons aged 60+ years, %	7.3	7.2	7.3
Muslim, %	76.3	85.5	72.0
Urban, %	3.0	11.0	3.5
Unions, n	12	10	10
Villages (wards), n	141	229	238
Health facilities, n			
UHC^b^	1	1	1
Community clinics	35	40	34
Literacy rate, %	52.9	53.9	47.3

a Data source: 2011 Bangladesh Census, Bangladesh Ministry of Health and Family Welfare facility registry [45,46].

b UHC: Upazila health complex.

### Ethical Considerations

This study was approved by the Institutional Review Board of James P Grant School of Public Health, BRAC University (reference number: IRB-16 November-23‐041). Written informed consent was obtained from all participants. Identifying information associated with the study participants will be kept confidential through unique identifying numbers on all paper forms and computer-based files to protect privacy and ensure confidentiality of data being collected. Participants received no compensation for participation.

### Interventions

#### Development of the Interventions

**Figure 2.**
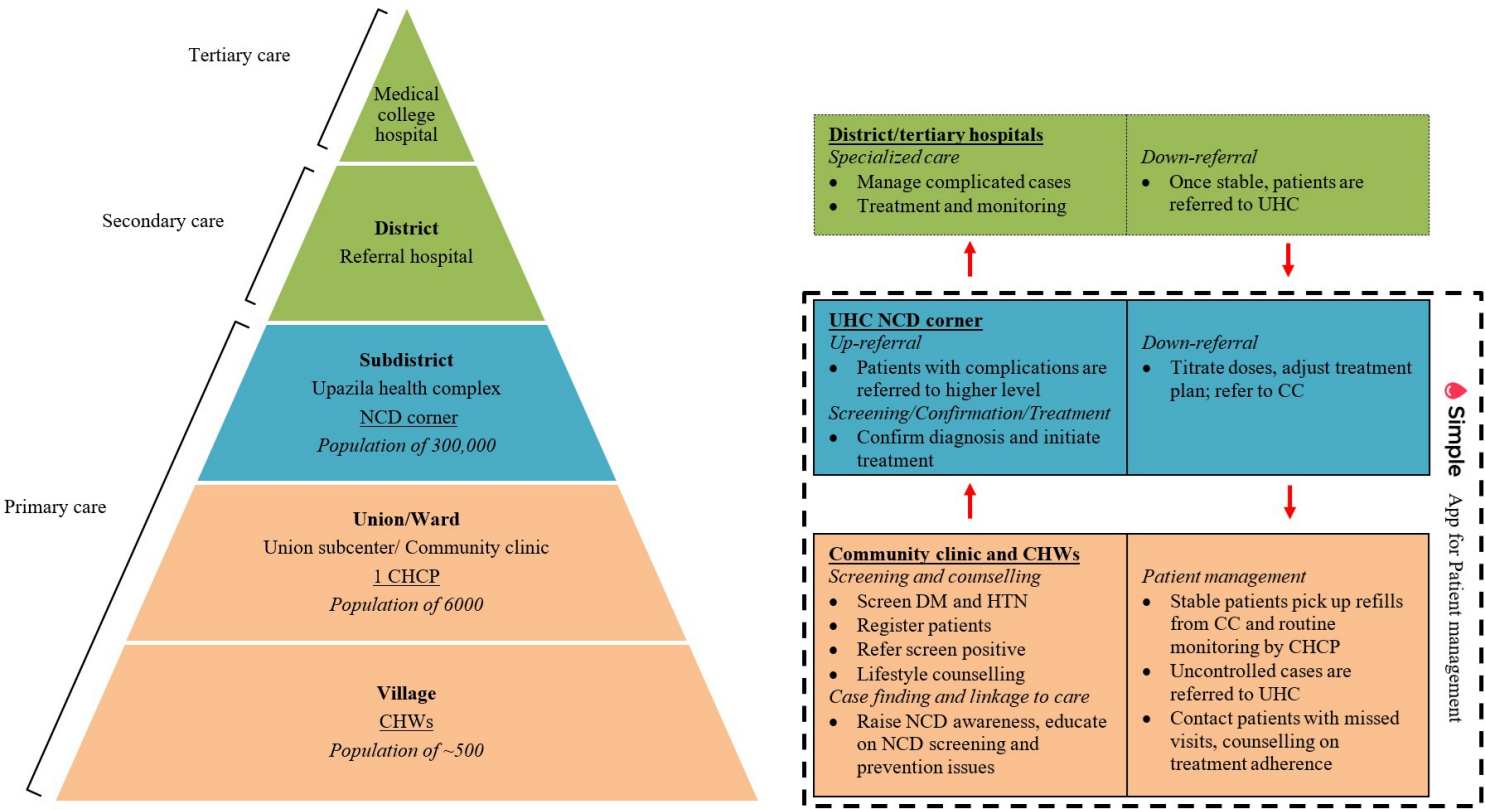
NCD service delivery hierarchy and decentralized care (adapted from Xie et al [26], which is published under Creative Commons Attribution 4.0 International License). CC: community clinic; CHCP: community health care provider; CHW: community health worker; NCD: noncommunicable disease; Tx: treatment; UHC: Upazila health complex.

Building on an assessment of hypertension and diabetes management barriers at the patient, provider, and health system levels [47-50], along with evidence from previous intervention studies and the updated UK Medical Research Council (MRC) guidance on developing and evaluating complex interventions [31-33,51], a multicomponent intervention package has been designed to increase access to primary care and to improve care quality and patient retention (Figure 2).

#### Multicomponent Decentralized Care

##### Digital Health for Patient Management

In alignment with the Bangladesh government recommendations, the Simple app is being used in primary care facilities for coordinated hypertension and diabetes management. The app supports prescription and continuity of care through unique patient IDs and enables functions including: (1) monitoring patient longitudinal BP and BG changes, prescription history, and prompting titration when indicated; (2) flagging overdue patients for action; and (3) generating performance reports on indicators such as patient enrollment and BP and BG control rates. A dedicated project assistant supports health workers with data entry and follow-up with overdue patients.

##### Decentralization With Task Sharing

Supported by the subdistrict NCD corner, 15 CCs were equipped to conduct screening, follow-ups, and medication refills. Implementation includes several core components: (1) providing regular trainings (every 6 months) for health care providers from the subdistrict NCD corner and CCs on team-based NCD care, (2) equipping CCs for routine care, and (3) providing regular supportive supervision by health officials. CCs received medical devices, Android tablets for record keeping, and essential medications.

##### Involvement of Community Health Workers

The intervention incorporates hypertension and diabetes management into community-based care through trained CHWs, leveraging evidence from successful LMIC programs [31-33,51-54]. CHWs perform 4 core functions: community screening and case identification, lifestyle counseling and health education, referral coordination, and patient follow-up. Capacity building includes an initial 6-day training conducted in June 2024, supplemented by biannual refresher trainings.

##### Supportive Monitoring and Supervision and Team-Based Care

The intervention incorporates a multitiered supportive supervision system to ensure quality service delivery. At the subdistrict level, NCD corners receive regular oversight from senior health administrators and medical professionals who address clinical management challenges, medication supply issues, and operational barriers. These supervisory visits are data-driven, using performance metrics generated by the Simple app dashboard to identify priority areas for improvement.

A cascading supervision framework extends to CCs, where subdistrict health officials conduct routine monitoring visits. Community health care providers (CHCPs) similarly provide ongoing supervision to CHWs in their catchment areas. To strengthen health system coordination, monthly meetings convene providers across levels to enhance collaboration, troubleshoot challenges, and optimize care processes.

Throughout the intervention period, dedicated technical support teams maintain system functionality by providing continuous assistance with the Simple app operations and troubleshooting.

##### Care Continuum Within the Multicomponent Decentralized Care

CHWs conduct targeted health promotion activities to increase NCD and their risk factors, with particular emphasis on encouraging adults aged ≥40 years to undergo screening at CCs. Adults detected with elevated BP or BG at CCs are referred to the subdistrict NCD corner for confirmation and care plan initiation if diagnosed. Patients with known hypertension and diabetes are referred to the NCD Corners for care plan initiation or resumption (for those who discontinued treatment).

At the subdistrict NCD corner, a structured clinical workflow ensures comprehensive patient management. Nurses perform detailed clinical assessments and maintain up-to-date patient records in the digital system. Physicians initiate care plans for new patients and decide if existing patients can be referred to CCs for routine follow-up and management according to the national guidelines (ie, having a controlled status in the three most recent consecutive visits). Prescriptions are guided by the Simple app BP/BG record and prescription history; treatment titration may be done when indicated.

At CC, CHCPs deliver essential NCD services for stable patients, comprising (1) routine clinical monitoring, (2) prescribed medication dispensing, and (3) lifestyle modification counseling. Patients maintaining treatment targets continue community-based management through scheduled follow-ups. For cases demonstrating suboptimal control or presenting with acute symptoms, CHCPs initiate immediate referral to the subdistrict NCD corner for physician evaluation and therapeutic change.

### Digital Health–Only Intervention

In the subdistrict (Parbatipur) with digital health–only intervention, initial training on the national protocols for hypertension and diabetes management and the Simple app in the NCD corner was conducted in July 2024. Biannual refresher training will be done throughout the implementation period. A project assistant is hired to help with the Simple app data entry and to follow up with overdue patients. CCs and CHWs are essentially not involved in care provision. Patient pathways remain the same as in usual care.

### Usual Care

The usual care subdistrict (Biral) receives biannual training on national protocols for hypertension and diabetes management. Existing usual care is being provided by the subdistrict NCD corner, including screening, treatment initiation, drug refill, and routine follow-up. CCs and CHWs are not involved in NCD care provision. The local government agreed to delay the rollout of the Simple app in the comparison subdistricts until the end of the study to avoid contamination.

### Effectiveness and Implementation Outcomes and Assessment

The primary outcome is the proportion of treated patients achieving or maintaining disease control according to national standards and WHO PEN (WHO Package of Essential Noncommunicable Diseases Interventions) protocols, with hypertension control defined as systolic/diastolic BP <140/90 mm Hg and glycemic control as fasting capillary glucose < 7.0 mmol/L or random capillary glucose <11.1 mmol/L. Effectiveness is being assessed primarily using data from repeated community-based surveys. Secondary outcomes of this study include hypertension and diabetes care cascade (ie, percentage of patients ever screened, percentage aware of their condition, and percentage on treatment), changes in health behaviors (eg, smoking cessation among hypertension and diabetes patients, and meeting recommended weekly physical activity level). To complement assessment of population-level changes using repeated community-based surveys, facility-level data will be extracted to evaluate changes in care quality at primary care facilities.

**Figure 3.**
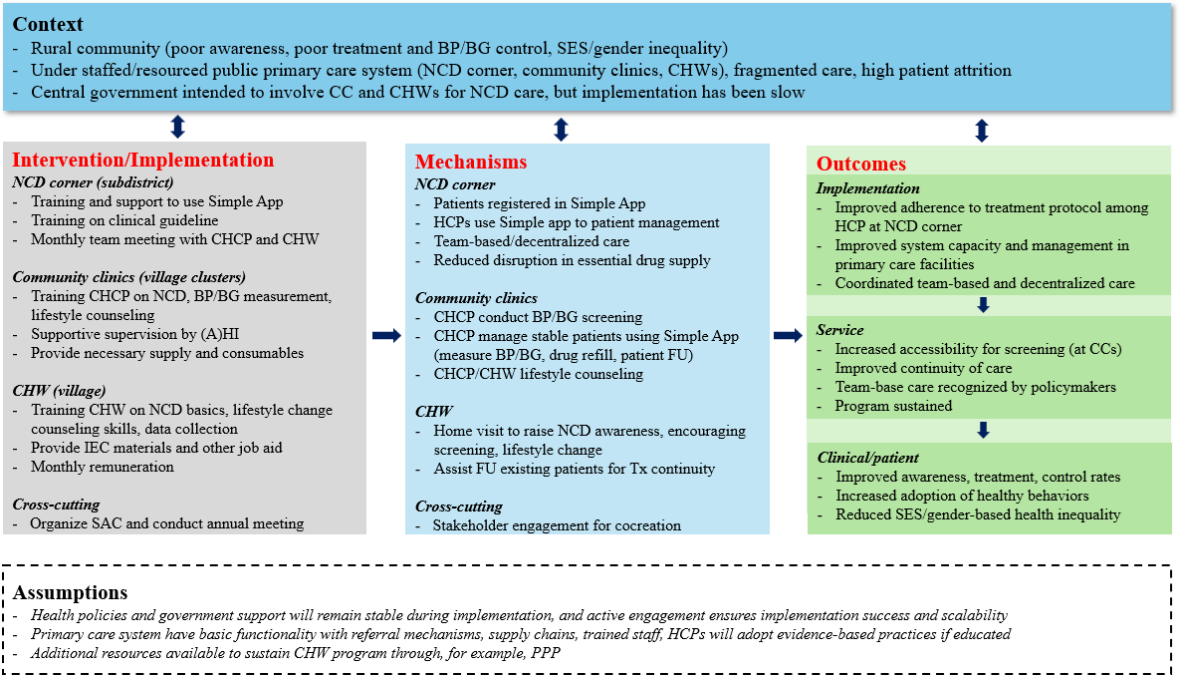
Program theory. (A)HI: assistant health inspector; BG: blood glucose; BP: blood pressure; CC: community clinic; CHCP: community health care provider; CHW: community health worker; FU: follow-up; HCP: health care provider; IEC: information, education, communication; NCD: noncommunicable disease; PPP: public-private partnership; SAC: stakeholder advisory committee; SES: socioeconomic status; Tx: treatment.

Specific aim 2 is evaluated by using the RE-AIM (reach, effectiveness, adoption, implementation, and maintenance) framework (Table 2) with patient assessments and stakeholder interviews, informed by previous studies [25,55]. Implementation fidelity and process evaluation are guided by the MRC guidelines on process evaluation of complex interventions [56] and the WHO’s NCD Facility-Based Monitoring Guidance [57]. The program theory is depicted in Figure 3. Data sources include training reports, prescription practice captured by the Simple app, facility records, and patient registries. The Simple app captures essential data related to patient background, prescriptions and titration, dates of follow-up visits, and longitudinal BP and BG records. Baseline qualitative data were collected from patients, health care providers, CHWs, and public health managers to inform the evaluation of barriers and enablers of implementing the interventions in the primary care system for improved NCD outcomes. Another round of qualitative data collection will be conducted upon completion of the interventions.

**Table 2.** Data collection plan for evaluation indicators.

RE-AIM^a^ domains or indicators	Operational definition	Data source	Instrument	Timeline, at months
Effectiveness				
Primary outcomes				
Percentage of patients with HTN^b^ who achieved BP^c^ control	BP <140/90 mm Hg	Community surveys	Omron HEM-7120	0, 12, 24
Percentage of patients with DM^d^ who achieved glycemic control	Fasting capillary glucose <7.0 mmol/L or random capillary glucose <11.1 mmol/L	Community surveys	Accu-Chek Instant	0, 12, 24
Secondary outcomes				
Percentage of adults with HTN aware of their condition	Ever been told by a health care provider that they have raised BP or HTN	Community surveys	Questionnaire	0, 12, 24
Percentage of adults with DM aware of their condition	Ever been told by a health care provider that they have diabetes	Community surveys	Questionnaire	0, 12, 24
Percentage of adults with diagnosed HTN on treatment	Currently taking medication for HTN, not including herbal or traditional remedy	Community surveys	Questionnaire	0, 12, 24
Percentage of adults with diagnosed DM on treatment	Taking medication for DM, not including herbal or traditional remedy	Community surveys	Questionnaire	0, 12, 24
Percentage of HTN and DM patients who quit smoking	Quit smoking is defined as not having smoked, even 1 or 2 puffs, during the past 6 months	Community surveys	Tobacco Use Questionnaire	0, 12, 24
Percentage of HTN and DM patients who met the recommended PA^e^ level	≥150 minutes MVPA ^f^ per week	Community surveys	WHO STEPS^g^	0, 12, 24
				
Reach				
Percentage of adults with HTN screened for HTN	Had a BP measurement in past 12 months	Community surveys	Questionnaire	0, 12, 24
Percentage of adults with DM screened for DM	Had a BG^h^ measurement in past 12 months	Community surveys	Questionnaire	0, 12, 24
Percentage of patients with HTN and DM receiving treatment from public PHC^i^ facilities	Currently receiving care from government PHC facilities	Community surveys	Questionnaire	0, 12, 24
Adoption				
Percentage of patients with HTN and DM registered in the Simple app	Information recorded in the Simple app, separated for CC^j^ and NCD^k^ corner	Simple app	Simple app	Quarterly
Percentage of CCs who conducted NCD screening	Screening ≥50 adults/month	Simple app	Simple app	Quarterly
Percentage of CCs who performed routine care for patients with NCD	Following up with ≥25 patients/month	Simple app	Simple app	Quarterly
Percentage of CHWs^l^ who conducted home visits	Home visits for ≥5 patients/month for lifestyle counseling and adherence support	CHW report	CHW report	Quarterly
Implementation				
Percentage of providers who participated in training and refresher	Participated in training on NCD management	Training report	Training registry	Biannually
Percentage of CCs with functional essential equipment and supply	BP/BG machines are calibrated, strips, and IEC^m^ materials available	Facility records	Facility report	Quarterly
Percentage of planned supportive supervision conducted	8 planned supervisions, separated by levels of PHC and CHW	Supervision report	Checklist	Quarterly
Maintenance				
Percentage of newly enrolled patients retained in care	LTFU^n^: ≥3 months late for the last scheduled visit, 6/12 months	Patient registry	Patient registry	12, 24, 30
CCs who performed routine care for NCD patients	Following up with ≥25 patients/month	Patient registry	Patient registry	30
NCD corner that used the Simple app for patient management	≥80% patient encounters recorded in the Simple app	Patient registry	Patient registry	30

a RE-AIM: reach, effectiveness, adoption, implementation, and maintenance framework.

b HTN: hypertension.

c BP: blood pressure.

d DM: diabetes mellitus.

e PA: physical activity.

f MVPA: moderate to vigorous physical activity.

g WHO STEPS: World Health Organization’s STEPwise approach to NCD risk factor surveillance.

h BG: blood glucose.

i PHC: primary health care.

j CC: community clinic.

k NCD: noncommunicable disease.

l CHW: community health worker.

m IEC: information, education, and communication.

n LTFU: lost to follow-up.

We anticipated some minor practical refinements and adaptations of the intervention components during the implementation process. Any refinements and the rationale for these are being documented for transparent reporting, guided by the program theory and updated MRC guidance.

For specific aim 3, the primary cost-effectiveness measure is the incremental cost per 1-percentage-point increase in the proportion of participants achieving control status over the 24-month intervention period. Detailed costs by input categories and steps in intervention components will be estimated. Both health system costs and patient costs will be compared between the intervention and usual care arms.

### Sampling Strategy and Power Calculation

Three community-based surveys are undertaken at months 0, 12, and 24. For each evaluation survey, an independent random sample of adults aged 40 years and above who are community residents in 3 study subdistricts is randomly selected through a multistage cluster sampling approach. We opted for a quasi-experimental design with repeated cross-sectional surveys, instead of a traditional randomized trial, to capture changes in the entire care continuum at the population level and to generate real-world evidence on effectiveness and implementation strategies for greater external validity [58,59]. A buffer zone between the 2 intervention subdistricts (ie, Chirirbandar and Parbatipur) was created to mitigate potential spillover effects between contiguous communities. Given the available resources and time constraints, we randomly select a total of 15 villages from each subdistrict. The villages are divided into segments of approximately 250 households without disrupting the boundaries of the villages. One segment per village is then randomly selected. Subsequently, a list of adults aged 40 years of age within each village of the selected segment is obtained. From each segment (cluster), an equal number of women and men ≥40 years of age are randomly selected by using systematic random sampling with the condition that no more than 1 adult male and 1 adult female is included from the same household.

The planned sample size was determined to be 6750 participants for each community survey, with an equal number of clusters per subdistrict (15 clusters per subdistrict, or 45 clusters total) and similar cluster sizes (150 participants: 75 females and 75 males per cluster). Assuming a prevalence of diagnosed hypertension of 40%, and 15% for diabetes among adults aged 40 years and above, based on South Asia Biobank [60] data (unpublished findings), we expected to collect information from 2700 individuals with hypertension and 1000 individuals with diabetes. Assuming a conservative intraclass correlation coefficient of 0.02 [25,61], and a 2-sided type I error rate of 5%, the survey has 80% power to detect a difference as small as 7 percentage points between the intervention and reference groups for BP control, and a 10 percentage point difference for glycemic control. Power calculations were performed using Stata 17 (Stata Inc.) power analysis for a clustered 2-sample proportions test [62]. The planned sample size for binary outcomes ensures sufficient power for continuous specifications of the outcomes [25,61,63].

### Data Collection, Management, Quality, and Security

Baseline community surveys used interviewer-administered questionnaires and physical examinations. The baseline questionnaire, an adapted version of the WHO STEPS (World Health Organization’s STEPwise approach to NCD risk factor surveillance) questionnaire, covers sociodemographic characteristics, comorbidities, medication use, and health behaviors. The questionnaire was translated into Bengali and piloted for clarity and digitized using Kobo Toolbox for data collection. BP was measured by research staff using Omron HEM-7120. Capillary BG after at least 8 hours of fasting was measured using Accu-Chek Instant (Roche Diabetes Care).

Facility-based data collection is being conducted via the Simple app by on-site staff. Demographic and NCD history, medication use, BP/BG measurements, and treatment dosages are being recorded. In usual care subdistricts, the same data are being collected through patient registry reviews. Additional facility-level data, such as medication availability, device functionality, and treatment success rates, are being collected for process evaluation. An acceptability and utility survey of the Simple app for NCD management will be administered to health care providers in intervention subdistricts at months 12 and 24. All providers involved in hypertension or diabetes care at NCD corners and CCs will be invited to participate.

All health care providers in primary care facilities who are directly involved in NCD care were approached for qualitative data collection at baseline. This included 15 doctors and nurses from 3 subdistrict NCD corners and 15 CHCPs from CCs. Additionally, 6 public health officials (2 from each subdistrict) were approached for an in-depth interview (IDI). Furthermore, 9 focus group discussions (FGDs) with patients living with hypertension, diabetes, or both conditions were conducted. For each FGD, 8‐12 patients were included. Participants were purposively selected to maximize the diversity of the sample on sociodemographic characteristics (eg, age, sex, religion, socioeconomic status, and geographic distance to health facilities) and NCD history (new and experienced). Qualitative data collection will be repeated by the end of the study with the same group of public health officials and NCD care providers. In cases of staff turnover, the ones who are in position at the time of data collection will be approached. Patient FGDs will be repeated with the same groups of participants. Moreover, all 15 CHWs will be approached for an FGD session. FGD/IDI guides were developed based on the WHO’s 7-domain framework of health care delivery [64], chronic care model [10], and prespecified program theory. Interviews will be audio-recorded and are expected to each take 30‐45 minutes to complete. Qualitative data collection will be performed at baseline and endline surveys.

The qualitative data collected from health administrators, clinicians, and CHWs in intervention subdistricts provide qualitative insights on barriers and facilitators to NCD care delivery, experiences with the Simple app, study participation, workflow restructuring, and teamwork with clinics and CHWs. Data collection at endline allows participants to reflect on changes in these aspects over time. Patient FGDs explore barriers and facilitators to care accessibility and perceptions of care quality, including specific intervention components like drug refills, CHW home visits, and CHCP follow-ups.

Hypertension- and diabetes-related treatment costs were collected for economic evaluation, including direct and indirect costs (eg, transportation, food, childcare, and lost work time) at baseline. Health service delivery costs, including staff, transportation, laboratory, training, utilities, and overheads, are being assessed using microcosting to track time and resources spent on activities [65]. Intervention costs account for the setting, target population, resources, and consumables such as medical supplies and overheads.

To protect privacy and ensure confidentiality of data being collected, identifying information associated with the study participants is being kept confidential through unique identifying numbers on all paper forms and computer-based files. This file linking names and study numbers is password-protected, only accessible to authorized study personnel. All electronic data are encrypted, password protected, and stored in secure computer networks. All study personnel were trained to follow standard protocols.

### Adverse Event Monitoring

Serious adverse events, including death, hospitalization, and other conditions that result in persistent or significant disability, were handled by medical professionals at the subdistrict NCD corner as per standard protocol. Health care providers at CC were required to send patients back to the subdistrict NCD corner for evaluation when persistent poor control of BP and BG is observed among patients managed at CCs. Adverse events were recorded by study personnel.

### Statistical Analysis Plan

Baseline characteristics of participants, including sociodemographic characteristics, medical history, anthropometrics, and lifestyle factors, will be compared between the 2 intervention and 1 control subdistricts using 1-way ANOVA or Rao-Scott χ^2^ tests. Analyses will be stratified by hypertension and diabetes status. A difference-in-difference estimate for hypertension and diabetes will be implemented with multivariate logistic regression analyses, which take the following form:


$$\log(p/1-p)=\beta_{0}+\beta_{1}{\;}^{*}Time\;+\beta_{2}{\;}^{*}Intervention\;+\beta_{3}{\;}^{*}[Time^{*}Intervention]+\beta_{4}{\;}^{*}Covariates+\varepsilon$$

The *β*_3_ coefficient captures the difference in change over time. Clustered standard errors at the village segment level will be specified to allow for intragroup correlation, relaxing the usual requirement that the observations within a cluster are independent [66]. Several individual- and area-level covariates (*β*_4_) will be included in the analytic models to mitigate confounding by potential different sample composition. These covariates will include age, sex, education, wealth, tobacco use, physical activity, self-reported fruit and vegetable intake, self-reported history of chronic disease (eg, CVD, chronic respiratory disease, cancer, and chronic kidney disease), and rural or urban status. Given that the evaluation follows a multistage process whereby village segment clusters are randomly sampled from each subdistrict, and individuals are sampled randomly from the village segment clusters, a clustering adjustment for standard error is necessary to avoid inflating the precision of the estimated intervention effect [67,68]. Marginal effects will be calculated to illustrate how the predicted probability (ie, the proportion of patients with controlled conditions) changes over time among the three groups. Potential heterogeneity of effects will be explored in subgroup analyses, defined by age, sex, religion, and socioeconomic status. Several sensitivity analyses will be performed to check the robustness of the findings to critical model assumptions and missing data. While the “parallel” assumption should be tested with repeated measures prior to the intervention, this was not feasible given the budget and other limitations. Instead, we are testing the assumption with a negative control in treatment or outcome. Second, we will test the robustness of the results to different modeling approaches to handle geographical clustering of participants and over time. Third, missing data patterns and potential mechanisms (ie, whether missing at random can be assumed) will be assessed. If missing data only affect a small proportion (ie, <5%) of the sample, listwise deletion will be done; otherwise, missing data will be handled with multiple imputation with chained equations if missing at random assumption is reasonable.

The facility-based cohort data collected longitudinally will capture patients’ treatment trajectory over the study period. Changes in the proportion of patients with a controlled condition will be analyzed with generalized estimating equation Poisson regression with robust variance [69,70]. Continuous changes in systolic BP and diastolic BP from baseline will be assessed with linear mixed effects models.

For qualitative data, dual Bengali-English language speakers on the study team will transcribe and translate the audio-recorded IDIs and FGDs into English. The English language version of the transcripts will then be coded and analyzed thematically using NVivo, a qualitative data software program developed by Lumivero. Qualitative data will allow the identification of specific barriers to and facilitators of interventions by exploring the experiences of patients and clinicians engaged in different models of NCD care delivery. Qualitative data analysis will be guided by prespecified program theory, the RE-AIM framework, and standard grounded theory to identify themes, build and apply codebooks, and describe thematic characteristics, patterns, and relationships [71,72].

For the economic evaluation, we will compare the costs and effects of intervention with the usual care group, both from financial and economic perspectives [73]. Financial cost per unit outcome will be calculated by dividing the total cost by the quantifiable unit of outcome (screening, treatment, retention in care, and control) for each of the three groups. Incremental cost-effectiveness ratios (ICERs) will be computed to compare the additional costs and effects of each intervention with the usual care. A nonparametric bootstrap with 1000 replications will be used to estimate 95% CIs around the point estimate of ICERs. All local currency (Bangladeshi Taka) costs will be converted to US dollars using the prevailing exchange rates and will be adjusted for inflation rates, discounted at 3%, and expressed in the 2025 US dollar present value terms. Sensitivity analysis will explore the robustness of the results to alternative probability distributions, time horizon, and uncertainty of key variables.

Descriptive statistics were reported for baseline characteristics of the sample stratified by subdistricts. Percentage estimates for key primary and secondary outcomes were age-standardized to the 2023 Bangladeshi population using age groups 40‐49, 50‐59, and ≥60 years, derived from the United Nations’ Population Division of the Department of Economic and Social Affairs World Population Prospects. We used a direct standardization approach using the STDIZE and STDWEIGHT Stata commands. The preliminary analyses were performed using Stata 18.0 (StataCorp Inc).

### Stakeholder Engagement

To ensure that the strategies developed are feasible, contextually appropriate, and sustainable in real-world settings, stakeholder groups (including government agencies, health care providers, and community representatives) have been actively engaged to enable cocreation. At the study design phase, input from relevant government agencies, major nongovernmental organizations, and community representatives was sought. During implementation, monthly meetings with subdistrict-level public health officials and CHWs were convened to facilitate bidirectional exchange of knowledge. As a member of the Bangladesh national committee for the revision of the national hypertension and diabetes protocol, the site principal investigator has shared findings with national policymakers. A stakeholder advisory group has been convened, with its first annual meeting planned for the end of 2025.

## Results

A baseline community survey was conducted between January and March 2024. The baseline data consist of a sample of 6849 adults aged 40 years and above randomly selected from 45 villages from 3 subdistricts (Biral, Parbatipur, and Chirirbandar) in Dinajpur district. Exclusion criteria included pregnancy, adults with terminal illness who have difficulty completing the survey or taking anthropometric measurements and physical examinations, and people who are unable to give consent. The sample was drawn with a 3-stage cluster sampling scheme. The first stage included a selection of 45 villages, which were selected stratified by subdistrict and proportional to population size. Stage 2 included the selection of one segment from each village, with each segment consisting of ~250 households. Household listing was done to create a sampling frame of male and female adults aged 40 years and above currently residing in the selected villages. A simple random sample of 88 male adults and 88 female adults was selected with the condition that not more than 1 male or female could be selected from the same household. The response rate was 86%.

The social and demographic characteristics of participants involved in the baseline survey are presented in Table 3. Overall, the mean age of the participants was 55.9 (SD 10.6) years; almost exactly 50.1% (3432/6849) were female. Forty percent of participants (2704/6849) had no formal schooling, and only 12.1% (829/6849) had secondary or higher education. The majority (6316/6849, 92.2%) of the sample was self-employed or worked as a homemaker. The majority of the sample was Muslim (5313/6849, 77.6%) and was currently married (5932/6849, 86.6%). Other than religion, employment, and wealth score, there was no statistical difference among the 3 subdistricts (arms) based on these sociodemographic characteristics.

**Table 3.** Sample characteristics of baseline community survey.

Variables	Total	Comparison groups			
		Usual care	Digital only	Multicomponent	*P* value
Participants, n (%)	6849 (100)	2262 (33.0)	2287 (33.6)	2300 (33.4)	
Age in years, mean (SD)	55.9 (10.6)	56.0 (10.4)	56.1 (10.9)	55.7 (10.6)	.37
Sex, n (%)					.96
Male	3417 (49.9)	1134 (50.1)	1143 (49.7)	1140 (49.8)	
Female	3432 (50.1)	1128 (49.9)	1157 (50.3)	1147 (50.2)	
Education attainment, n (%)					.73
No formal schooling	2704 (39.5)	916 (40.5)	897 (39.0)	891 (39.0)	
Primary	3319 (48.4)	1075 (47.5)	1116 (48.5)	1125 (49.2)	
Secondary or higher	829 (12.1)	271 (12.0)	287 (12.5)	271 (11.8)	
Marital status, n (%)					.58
Currently married	5932 (86.6)	1954 (86.4)	1983 (86.2)	1995 (87.2)	
Widowed or other	917 (13.4)	308 (13.6)	317 (13.8)	292 (12.8)	
Religion, n (%)					<.001
Muslim	5313 (77.6)	1741 (77.0)	1868 (81.2)	1704 (74.5)	
Non-Muslim	1536 (22.4)	521 (23.0)	432 (18.8)	583 (25.5)	
Employment, n (%)					<.001
Employed or retired	212 (3.1)	69 (3.1)	72 (3.1)	71 (3.1)	
Self-employed or homemaker	6316 (92.2)	2093 (92.5)	2082 (90.6)	2141 (93.6)	
Unemployed	319 (4.7)	100 (4.1)	144 (6.3)	75 (3.3)	
Wealth score, n (%)					.004
Low	2261 (33.0)	736 (32.5)	761 (33.1)	764 (33.4)	
Medium	2270 (33.1)	703 (31.1)	768 (33.4)	799 (34.9)	
High	2318 (33.8)	823 (36.4)	771 (33.5)	724 (31.7)	
BMI (kg/m^2^) category, n (%)					.30
<18.5	911 (13.3)	306 (13.5)	321 (14.0)	284 (12.4)	
18.5‐22.9	3067 (44.8)	1041 (46.0)	1024 (44.5)	1002 (43.8)	
23.0‐27.4	2185 (31.9)	701 (31.0)	731 (31.8)	753 (32.9)	
≥27.5	686 (10.0)	214 (9.5)	224 (9.7)	248 (10.8)	
Elevated WC^a^, n (%)	2583 (37.7)	827 (36.6)	878 (38.2)	878 (38.4)	.45
Mental health^b^, n (%)					
Depression	666 (9.7)	252 (11.1)	191 (8.3)	223 (9.8)	.005
Anxiety	361 (5.3)	161 (7.1)	80 (3.5)	120 (5.2)	<.001
Family history, n (%)					
Premature CVD^c^	702 (10.2)	249 (11.0)	252 (11.0)	201 (8.8)	.02
Diabetes	1155 (16.9)	399 (17.6)	355 (15.4)	401 (17.5)	.09
Self-reported medical history, n (%)					
CVD	202 (2.9)	54 (2.4)	70 (3.0)	78 (3.4)	.12
COPD^d^/asthma	307 (4.5)	111 (4.9)	125 (5.4)	71 (3.1)	<.001
Other NCD^e^	231 (3.4)	75 (3.3)	74 (3.2)	82 (3.6)	.76
Hypertension, n (%)	2690 (39.3)	876 (38.7)	959 (41.7)	855 (37.4)	.01
Diabetes, n (%)	967 (14.1)	329 (14.5)	298 (13.0)	340 (14.9)	.14

a WC: waist circumference.

b Depression and anxiety were assessed using PHQ-2 and GAD-2, respectively, with a cutoff point of ≥3 as indicating these mental health symptoms.

c CVD: cardiovascular disease.

d COPD: chronic obstructive pulmonary disease.

e NCD: noncommunicable disease.

Among all participants, the prevalence of hypertension, diabetes, overweight and obesity, and self-reported CVD were 39.3% (2690/6849), 14.1% (967/6849), 41.9% (2871/6849), and 2.9% (202/6849), respectively. Ten percent (702/6849) of the participants had a family history of premature CVD, and 16.9% (1155/6849) had a family history of diabetes. Across the 3 arms (subdistricts), the digital intervention arm appeared to have a higher prevalence of hypertension compared with the multicomponent and the usual care arms (855/2300, 41.7%; 959/2287, 37.4%; and 876/2262, 38.7%; respectively).

The baseline age-standardized BP control rate among participants with hypertension was 10.2%, while baseline glycemic control among participants with diabetes was 14.9% (Table 4). There were slight differences across the 3 subdistricts. For secondary outcomes, age-standardized awareness and treatment rates for hypertension were 35.3% and 23.0%, respectively, and 60.7% and 33.9% for diabetes, respectively. Among participants living with hypertension, diabetes, or both conditions, 47.1% met the physical activity level recommended by WHO, and 48.5% had quit smoking.

**Table 4.** Outcome measures at baseline.^a^

Outcomes	Total, n (%)	Comparison groups, n (%)			
		Usual care	Digital only	Multicomponent	*P* value
Primary outcomes					
Achieved blood pressure control^b^	263 (10.2)	67 (8.5)	80 (8.8)	116 (13.4)	.003
Achieved glycemic control^c^	154 (14.9)	72 (20.0)	36 (11.0)	46 (13.5)	.001
Secondary outcomes					
Awareness of hypertension status^b^	942 (35.3)	287 (33.4)	324 (34.2)	331 (38.6)	.06
On treatment for hypertension^b^	635 (23.0)	166 (19.1)	212 (21.3)	257 (29.0)	<.001
Awareness of diabetes status^c^	591 (60.7)	215 (65.3)	169 (56.5)	207 (60.2)	.09
On treatment for diabetes	334 (33.9)	96 (27.3)	105 (34.5)	133 (39.1)	.03
Patients with hypertension/diabetes who met physical activity target^d^	1461 (47.1)	469 (45.9)	441 (41.2)	551 (54.6)	<.001
Patients with hypertension/diabetes who quit smoking^e^	579 (48.5)	189 (48.2)	205 (52.7)	185 (45.4)	.05

a Percentage estimates are age-standardized to the 2023 Bangladeshi population using age groups 40‐49, 50‐59, and ≥60 years, derived from the United Nations’ Population Division of the Department of Economic and Social Affairs World Population Prospects.

b The number of participants with hypertension was 2690 overall (867, 959, and 855 for usual care, digital-only, and multicomponent arms, respectively).

c The number of participants with diabetes was 967 overall (329, 298, and 340 for usual care, digital-only, and multicomponent arms, respectively).

d The number of participants with hypertension, diabetes, or both conditions was 3137 overall (1021, 1101, and 1015 for usual care, digital-only, and multicomponent arms, respectively).

e The number of participants with hypertension, diabetes, or both conditions who ever smoked was 957 overall (328, 309, and 320 for usual care, digital-only, and multicomponent arms, respectively).

## Discussion

Driven by population aging, rapid urbanization, and the globalization of unhealthy lifestyles, the burden of NCDs is rapidly increasing in LMICs [2,74,75]. However, access to quality care remains limited in LMICs, particularly in rural areas. Awareness, diagnosis, treatment, and control rates of hypertension and diabetes are low. Strengthening primary care to address NCDs requires novel approaches to address complex barriers at the health system, provider, and patient levels [12]. Several features of NCDs, including shared lifestyle risk factors, comorbidity, and chronicity, necessitate a shifting of care organization from episodic care toward a long-term integrated approach to prevention, diagnosis, and treatment across overlapping conditions [12]. A well-coordinated team-based model of care is critical for the care continuity necessary to achieve sustained control of chronic conditions [29,76]. Digital technologies offer the potential to tackle health system challenges in LMICs, improve access to and quality of health care, and reduce health system costs [77]. The emergence of the COVID-19 pandemic has intensified the need for digital technologies to support decentralized NCD care [78,79]. However, few digital interventions have demonstrated effectiveness in NCD care [28,30,80]. Progress toward successful implementation and scaling up of digital innovations for primary care delivery in LMIC settings remains limited and requires collaborative research and development efforts between health system stakeholders and technology communities to address holistically.

This study is among the first to evaluate the effectiveness, cost-effectiveness, and implementation strategy of a digital technology-supported decentralized primary care model for integrated hypertension and diabetes management in an LMIC context [24,25]. With a combination of repeated cross-sectional community surveys, routinely collected facility-based longitudinal data, qualitative data collection, and a cost-effectiveness evaluation, this study is expected to provide rich implementation and effectiveness data on a multicomponent digital technology-supported decentralized intervention for integrated hypertension and diabetes care. The findings from the study will provide valuable insights for the Simple app developers and the larger global health technology community, supporting their efforts to develop effective digital solutions to address NCD challenges.

The baseline community survey has been completed with a high response rate and excellent data completion rates. At the time of writing this paper, initial training of health care providers and managers has been completed, the Simple app has been successfully deployed, and CCs have been equipped to provide hypertension and diabetes screening, referral, and management. Facility-based data collection has been set up. Data analysis on the baseline community survey and facility-based survey is ongoing. Stakeholders have been actively engaged, and initial findings have been shared with relevant government agencies.

Designed to generate rich process and implementation data, the study is limited in the number of primary care facilities involved and geographical representativeness. Thus, the results will need to be interpreted in relation to the specific regional socioeconomic context. Nevertheless, our implementation assessment results will shed light on the transportability of the interventions in different contexts, which might include density of primary facilities, density and information technology knowledge of health care providers, availability of CHWs, internet connection, etc. Furthermore, unlike a randomized controlled trial, the quasi-experimental approach relies on a strong “common trends” assumption to establish a counterfactual. Without randomization, causal inferences are difficult to establish. Nevertheless, we have collected extensive data on potential confounding variables and planned extensive sensitivity analyses to evaluate potential biases as previously discussed.

As the government of Bangladesh is taking more steps to mitigate the increasing burden of NCDs and to achieve the Sustainable Development Goals, the evidence generated from the proposed study will be directly relevant for policymaking and programmatic efforts for NCD prevention and management in Bangladesh. The implementation and cost-effectiveness data may be particularly important to inform the scalability and sustainability of the interventions. The findings will likely be internationally relevant, as many LMICs share similar challenges as Bangladesh regarding NCD prevention and control.

## Abbreviations

BG: blood glucose
BP: blood pressure
CC: community clinic
CHCP: community health care provider
CHW: community health worker
CVD: cardiovascular disease
FGD: focus group discussion
ICER: incremental cost-effectiveness ratio
IDI: in-depth interview
LMIC: low- and middle-income country
MRC: UK Medical Research Council
NCD: noncommunicable disease
RE-AIM: reach, effectiveness, adoption, implementation, and maintenance framework
WHO: World Health Organization
WHO PEN: WHO Package of Essential Noncommunicable Diseases Interventions
WHO STEPS: World Health Organization’s STEPwise approach to NCD risk factor surveillance
